# Large-scale sports events, sports gambling market and promotion risk management: Theoretical model and case analysis based on option hedging theory

**DOI:** 10.1371/journal.pone.0286990

**Published:** 2023-06-21

**Authors:** Qi-an Chen, Xu Zhao, Guohong Zhang

**Affiliations:** 1 School of Economics and Business Administration, Chongqing University, Chongqing City, Sichuan Province, China; 2 Surrey International Institute, Dongbei University of Finance and Economics School, Jianshan Street, Shahekou District, Dalian City, Liaoning Province, China; University of Murcia: Universidad de Murcia, SPAIN

## Abstract

Large-scale sports events have become a good opportunity for major enterprises to promote due to their high social attention; however, they also force enterprises to confront the risks of uncertainty and extreme loss. During the 2018 Russia World Cup, Vatti Co., Ltd.’s promotion activity “If France Wins, Get a Full Refund” suffered double losses economically and reputationally due to France’s victory and the company’s failure to fulfill its promise. This paper uses option hedging theory, and the risk management tools to construct a risk management model. Case analysis and program improvement were carried out. The research results show that using the winning odds can effectively control the risks. Companies should determine their promotion plan based on sale returns and the maximum implicit income generated by promotional activities. The research paper opens a new field using derivative financial instruments to control corporate promotion risks.

## Introduction

Large-scale sports events have become a good opportunity for major enterprises to promote sales because of the social attention they attract; however, using large-scale sports events for promotion also faces the risk of extreme losses due to the uncertainty of sports competition results and the quantity of sales. During the 2018 Russia World Cup, the promotion activity “If France Wins, Get a Full Refund” launched by Vatti Co., Ltd. suffered double losses, economically and reputationally, due to France’s victory and the company’s failure to fulfill its promotion promise. Risk management and the avoidance of extreme losses in the promotion of large-scale sports events has become an important issue for enterprises. During large-scale sports events, world’s major gambling companies generally set odds on the competition’s results for the public to bet on, providing risk management tools from the financial derivatives market for companies to control the risk of uncertainty and extreme losses during promotional activities. The use of derivative financial instruments and risk hedging theories to effectively control company risk in large-scale sports events to carry out promotional activities is a new area of research and is urgently needed in corporate marketing practice and academic circles.

The development of option goes back a long period, since Sharp (1964) [1], Lintner (1965) [2] and Mossin (1966) [3]. Generally speaking, option hedging is an investment method that uses option trading to offset other investment risks. It can reduce business risks while still making profits on the investment. A common hedging has two trades which are correlated, in opposite directions, in equal quantities, and break even. Option hedging is divided into static hedging and dynamic hedging. Static hedging refers to the establishment of hedging positions and keep the position unchanged until the option expires, so that the holders do not need to adjust positions by observing market changes. The other one is dynamic hedging, referring to adjusting the hedging position before the option expiration to make the risk of the whole portfolio within the investor’s control. The most common dynamic hedging is Delta hedging.

As market competition has intensified, there has been a sharp rise in corporate operating risk. In order to guarantee the sustainable and stable development of enterprises, reduce corporate value losses and increase the value of shareholders’ equity, risk management should be an important part of corporate operating decisions [4], incorporated into the top-level system design of corporate business strategy [5]. At the same time, the fast-developing financial derivatives market has provided effective tools for companies to prevent and control operational risks [6], and the rational utilization of derivative financial instruments has been able to hedge and control unnecessary risks faced by companies [7]. For example, options such as common financial derivatives have been used for hedging and controlling various risks faced by enterprises in business operations [8].

In general, the purpose of enterprises using derivative financial instruments to hedge their operating risks is to reduce uncertainty and extreme loss risks in business operations. However, motivations differ amongst enterprises in the use of derivative financial instruments to hedge operating risks. The main purpose of most business operators in using financial derivatives has been to reduce operating risk and the volatility of cash flow and earnings [9], but there are also companies using financial derivatives to hedge their operating risks while also focusing on the corresponding income obtained by risk-taking [10]. The main purpose of large Australian listed companies using financial derivatives was to reduce the cost of financial distress and increase corporate value [11]. However, Australian extractive companies have regarded the reduction of commodity and exchange rate risks through use of derivative financial instruments as their main purpose [12]. Large UK non-financial companies and non-financial listed companies have been more active in the use of financial derivatives, with the purpose of managing the risks arising from cash flow fluctuations [13].

In terms of the effect of enterprises using financial derivatives to hedge their operating risks, Han Liyan and Zhang Xiaolei (2009) [14] found that some state-owned enterprises in our country suffered great losses in financial derivatives transactions without the effect of reducing their operating risks but believed this to be caused by the failure of internal control, absence of external supervision, and the oversight of the industry cycle. The losses mainly came from speculation rather than hedging. Fauver and Naranjo (2010) [15] conducted a study of 1,746 companies headquartered in the United States and found that the use of derivatives was negatively correlated with corporate value. They attributed this result to supervision problems and agency issues. Chen et al. (2010) [16] believed that it was conditional for companies using financial derivatives to hedge their business risks to produce good results. The effect of construction companies using financial derivatives to avoid financial risk depended on trading skills and forecast accuracy. Except for the few examples mentioned above, most of the existing relevant research has documented that the use of financial derivatives to hedge business risks would achieve the purpose of reducing risks and increasing value. Jin and Jordan (2006) [17] studied the hedging behavior of the financial derivatives of 119 oil and gas producers in the United States and found that hedging could reduce the sensitivity of a company’s stock price to petroleum and natural gas prices. Guo Fei and Wang Xiaoping wang (2009) [18] believed that a correct hedging strategy in a company could reduce the volatility of cash flow and reduce the likelihood of a fatal blow to the company from small probability events. Nguyen and Faff (2010) [19] took Australian listed companies as a sample and showed that the use of options to hedge business risks is not harmful to corporate value. Aretz and Bartram (2010) [20] considered that in case of deficiencies in the actual capital market, companies can create value for shareholders by using derivative financial instruments to hedge their operating risks. Bartram et al. (2011) [21] studied a sample of non-financial companies in 47 countries and found that the use of financial derivatives by companies could reduce total and systemic risk, while increasing corporate value. Guo Fei’s (2012) [22] study of 968 multinational companies in China indicated that the use of foreign exchange derivatives by Chinese multinational companies to hedge the exchange rate risk could bring a 10% value premium to companies. Sileo (2013) [23], after studying ski resort enterprises with high business risks, believed that the correct hedging of meteorological risks was a key factor in combating meteorological uncertainty. Zhou and Wang (2013) [24] found that after using derivatives to hedge the risk of adverse exchange rate changes, the risk exposure of UK non-financial companies decreased. Panaretou (2014) [25] studied British companies as the sample and found that hedging premiums had outstanding economic significance for users of foreign exchange derivatives. Paligorova and Staskow (2014) [26] studied listed companies using derivatives in Canadian companies, finding that using derivatives to hedge risks increased the scale and profits of companies and decreased the volatility of earnings. Chaudhry et al. (2014) [27] conducted a study on 75 non-financial listed companies on the Karachi Stock Exchange and found that companies could obtain scale economy by using derivatives. Zhang Qianhe (2014) [28] studied the behavior of enterprises using commodity futures for hedging and speculation and found that hedging could stabilize the business performance of enterprises, while speculation increased the volatility of corporate performance. Lau (2016) [29] found that companies with lower operating profit margins were more inclined to use derivatives to hedge the impact of financial risks. Furthermore, companies that use financial derivatives to hedge their operating risks were likely to increase their return on assets.

When companies used derivative financial instruments to hedge their business risks, they were often affected by various factors. Bartram et al. (2009) [30] investigated the use of derivative financial instruments in 7,319 companies from 50 countries and found that the company’s current assets, dividend policy, financial leverage, and hedging operation methods all affected the company’s decision making in the use of financial derivatives. International hotel accommodation industry companies mainly used interest rate swaps and options to manage interest rate risk. The main factors affecting their use of these financial derivatives to make risk hedging decisions included company size, foreign sales ratio, cash flow fluctuations, financial distress costs, underinvestment costs, and management risk avoidance requirements [31]. From the perspective of companies in different countries and regions, company size, return on assets, leverage ratio and foreign sales were important factors affecting Australian companies’ use of financial derivatives to manage and control operational risks [32]. Leverage ratio and company size played a very prominent role in deciding to use financial derivatives in Australian industrial and mining companies [33]. Commonly used derivatives in Australian extractive companies were forward interest rate agreements and options. Company size as well as financial risk also played a significant role in decision-making concerning the use of financial derivatives [12]. Key factors influencing the use of financial derivatives to hedge business risks by companies listed on the Taiwan Stock Exchange of China were company size, the proportion of exports, and the proportion of long-term debt to total debt [34]. Taiwan listed companies with a larger scale had more growth investment opportunities, and the higher cost of the financial crisis tended to use financial derivatives for hedging [35]. The use rate of financial derivatives by Peruvian companies has been relatively low, with the degree of training in market supervision and financial derivatives expertise the main factor leading to the low use rate of financial derivatives [36]. The size of the company, the relationship between the company and the bank, the participation of the company’s foreign business activities, and the price-to-book ratio could all have an important impact on the decision-making of Indonesian companies in the use of financial derivatives [37]. Malaysian companies that use foreign exchange derivatives generally have higher overseas sales and growth opportunities [38]. Korean companies with greater exports and foreign currency debts, and greater exposure to exchange rate risks were more likely to use currency derivative instruments to hedge their operating risk [39]. In addition, factors such as the level of professional knowledge of operators, the degree of information asymmetry, levels of national corruption, and the concentration of corporate equity also had an impact on the use of financial derivatives to manage and control business risk decisions. In a survey of 61 ski lift operators, Bank and Wiesner (2011) [40] found that most of the operators were aware of the risks to business operations caused by weather changes, but due to lack of relevant expertise and awareness, few operators considered using weather derivatives as method to offset potential losses. Lin and Lin (2012) [41] found that companies with moderate information asymmetry had a greater probability of using derivatives for hedging, while companies with higher or lower information asymmetry were more inclined to speculate. Kim et al. (2017) [42] studied 881 non-financial companies in eight East Asian countries and regions and found that the use of corporate derivatives had a certain relationship with the degree of corruption. Domestic and host countries with low levels of corruption would benefit from prompt companies to use more derivatives. Butt et al. (2018) [43] used a sample of 101 non-financial listed companies on the Pakistan Stock Exchange to study the impact of equity concentration on the use of corporate financial derivatives and found that while corporate executives may use financial derivatives to increase stock value, the major shareholders were unlikely to use derivatives to hedge risks for their own interests.

Based on the above analysis, it can be seen that the although the existing relevant research literature has conducted lots of studies on the purpose, effects, and influencing factors of enterprises using derivative financial instruments to hedge business risks, and there are many valuable and innovative research results. However, there is little discussion in the literature of the uncertainty risk and extreme loss risk management strategies of companies using large-scale sports events to carry out promotional activities based on derivative financial instruments and risk hedging theory. Neither is there a focus on designing an effective risk management plan that could be used in the promotion of the Vatti Co., Ltd. 2018 World Cup “If France Wins, Get a Full Refund” strategy. This research seeks to address this gap, firstly through an in-depth investigation of corporate promotional behavior and gambling market behavior characteristics during large-scale sports events, using option hedging theory to construct a risk management model for companies to carry out promotional activities through large-scale sports events. Based on the results of the theoretical model, the management and control strategies of uncertainty risk and extreme loss risk are designed for enterprises to carry out promotional activities with the help of large-scale sports events. Secondly, through analyzing the process of Vatti Co., Ltd. in its 2018 World Cup “If France Wins, Get a Full Refund” promotion activity, as well as the theoretical model results, the risk control scheme of the 2018 World Cup “If France Wins, Get a Full Refund” of the company was improved and designed. In summary, based on deep analysis of the company’s use of large-scale sports event promotion plans and processes, and the sports gambling market setting odds on sports events results, this paper uses option hedging theory and the risk management tools provided by the sports gambling market to construct a risk control model for companies using large-scale sports events for promotion campaigns. Case analysis and program improvement were carried out on the promotion activities of Vatti’s “If France Wins, Get a Full Refund” campaign.

## Assumptions and procedure description

For ease of modeling and description we take the following reasonable assumptions:

(1) Supposing the time interval of a large-scale sporting event G, such as the 2018 Russia World Cup [t1, T], is divided into two stages: non-elimination [t1, t2] and elimination [t2, T], t1 < t2 < T; each participant may be eliminated at the end of the non-elimination stage.
(2) A certain company (such as Vatti shares) launches the following promotional activities before opening the sports event (at time t0). For consumers who purchase the company’s designated product or product portfolio (during t0, t2), if a particular participant P (such as the French team) wins the championship, the company should refund the purchase price to the consumer based on the purchase invoice and the refund rate of r, where 0 ≤ r ≤ 1. When r = 1 the company should fully refund, and when r = 0 the company should not refund.
(3) Assume that the winning odds offered by the bookmaker for participant P at t0 is A0, A0 > 1. Usually the gambling company sets the winning odds for the contestants based on the probability of winning the championship. The greater the probability of winning, the lower the winning odds set by the gambling company. There is an anti-correlation relationship between the winning odds and the winning probability. At the same time, to ensure that the bookmaker can obtain excess expected returns, the company often adds an odds reduction factor α when setting the winning odds. Without loss of generality, suppose that the probability of the contestant P winning the championship at t0 is $p_{0}=\frac{1}{\alpha A_{0}}$, α > 1.
(4) The promotion of the designated product during the promotion period [t0, t2] is positively correlated with the refund rate (r) and the winning probability (p0) of participant P. Furthermore, when the refund rate r = 0 (equivalent to the company not carrying out promotional activities) or the contestant P definitely loses the championship (p0 = 0), it should not stimulate consumers to buy more designated products. At this point the promotion amount of the designated product is 0, and the total sales Q is equal to the sales of Q0 with no promotion. In general, suppose that the total sales of the designated product during the promotion period [t0, t2] of the company is

$$Q=Q_{0}+brp_{0}$$ (1)

Here b is the maximum promotion amount when the contestant P wins the championship (p0 = 1) and the company fully refunds (r = 1). It is equivalent to the company giving the designated product to consumers for free, so the amount of demand depends on the maximum amount of funds that consumers could raise during the promotion period [t0, t2] and the maximum production capacity for the designated products. The promotion amount b is often a very large value.

(5) Assuming that the return on sales on designated products is s, its total profit and promotion profit during the promotion period [t0, t2] can be expressed as *Q*∙*s* and (*Q*−*Q*_0_)∙*s*, respectively.
(6) The promotion activities may not only increase the sales volume of designated products during the promotion period and obtain obvious economic benefits, but also may increase the company’s social reputation and brand value through promotion, gaining implicit benefits. In the practice of sales promotion, companies may also lose part of their explicit economic gains by making concessions to consumers during the promotion activities to enhance social visibility and brand value. Obviously, the higher the refund rate and the more concessions given to consumers, the greater the social reputation for the company. In general, assuming the implicit income obtained by the company through promotional activities is

V=dr (2)

where d represents the maximum implicit income that a company obtains with the full refund and is also the maximum promotional expense that the company is willing to pay. It can be regarded as the maximum promotional direct economic loss that the company can bear d > 0.

## Module building and analysis

### Option description and risk analysis of companies using large-scale sports events to carry out promotional activities

The company utilizes the sporting event G to carry out the above promotion activities, equivalent to giving a free call option to the consumer when selling the designated product in the time interval [t0, t2]. The company and the consumers are the creator and holder of the call option, respectively. If contestant P wins the championship, then the consumer exercises the right, and the company should fulfil the contract. If contestant P loses the championship, then the consumer as the option holder does not exercise the right, and the company as the option creator is not required to fulfil the refund obligation. Therefore, the option (denoted as OP1) can be expressed as follows:

Trading venue: the company’s designated product sales market (including online and offline markets).
Subject of Option Contract: participant P.
Option contract exercise date: a period after the end of sports event G.
Exercise price: contestant P wins.
Contract type: call option (Participant P wins the championship to go up, and vice versa).
Option fee: free, that is, consumers get free call options from the company by purchasing designated products.

Based on the actual situation of corporate promotional activities and the characteristics of options, the following analysis can be made of the corporate and consumer benefits of promotional activities:

(1) Under the condition that contestant P losses the championship and the consumer bought the designated product without any additional losses, the enterprise does not need to refund because the consumer does not exercise the right. The promotion’s economic profit and implicit profit (increased by social awareness and brand value) are (*Q*−*Q*_0_)*s* and dr, and the total income is

$${TRN}_{0}=(Q-Q_{0})s+dr$$ (3)

(2) If P wins the championship, consumers pay for the designated product, as well as receiving a refund of Qr; the enterprise should perform the refund obligation due to the consumers and refund Qr to them. The promotion’s economic profit and implicit profit are (*Q*−*Q*_0_)*s*−*Qr* and dr, respectively, and the total income is

$${TRY}_{0}=(Q-Q_{0})s-Qr+dr$$ (4)

Obviously, the company faces two risks in this promotion: one is the uncertainty risk caused by whether the contestant P wins the championship, and the other is the extreme loss risk if contestant P wins the championship.

In fact, under the condition that contestant P wins the championship, ${TRY}_{0}=[-bp_{0}(r-s)-Q_{0}+d]r$. When the refund rate r in promotional activities is relatively high, its return on sales s is relatively low (less than the refund rate r), and the participant P is more likely to win the championship, the company could suffer great economic loss(*bp*_0_(*r*−*s*)*r*+*Q*_0_*r*) due to the huge refunds. When the maximum implicit income (increasing social visibility and brand value) d in the promotion activities is relatively small,(*d*<*bp*_0_(*r*−*s*)+*Q*_0_) the increasing social visibility and brand value cannot recover the direct economic loss caused by the promotion activities. This leads to the company not only being unable to increase its operating performance through promotion activities, but also possibly having a negative impact on its development prospects.

### Analysis of companies’ option hedging trading in the sports gambling market

In order to avoid or reduce the uncertainty risk and the extreme loss risk under the condition of participant P winning the championship, the company should bet $\frac{Qr}{A_{0}}$ with the odds A0, set by the gambling company at t0. This is equivalent to a company buying a call option for contestant P to win the championship in the betting market. If contestant P wins the championship, the company should exercise the right, and the betting company should perform the contract and pay the bet amount Qr to the company at the odds. If participant P loses the championship, then the company does not exercise the right and loses the betting capital $\frac{Qr}{A_{0}}$. The betting company fails to fulfill its obligations and earns the betting capital. The option (denoted as OP2) can be expressed as follows:

Trading venue: sports gambling market.
Subject of Option Contract: participant P.
Option contract exercise date: a period after the end of sporting event G.
Exercise price: contestant P wins the championship.
Contract type: call option (participant P wins the championship to go up, and vice versa).

$$Option\;fee:\;\frac{Qr}{A_{0}}$$

### Decisions on corporate promotional activities based on option hedging transactions

Based on the above options OP1 and OP2, the income of the enterprise under the condition of participant P losing or winning the championship can be calculated, respectively.

In the case of contestant P not winning the championship, the total income (including the promotion’s economic income and implicit income) obtained by the company in the product market is $(Q-Q_{0})s+dr$, and the economic income obtained in the gambling market is $-\frac{Qr}{A_{0}}$, its comprehensive total income is

$$TRN=(Q-Q_{0})s-\frac{Qr}{A_{0}}+dr$$ (5)

In the case of contestant P winning the championship, the total income obtained by the company in the product market is (*Q*−*Q*_0_)*s*−*Qr*+*dr*, the economic income obtained in the gaming market is $-\frac{Qr}{A_{0}}+Qr$ and its comprehensive total income is

$$TRY=(Q-Q_{0})s-Qr-\frac{Qr}{A_{0}}+Qr+dr=(Q-Q_{0})s-\frac{Qr}{A_{0}}+dr$$ (6)

It can be seen from Eqs (5) and (6) that after a company uses the sports betting market for option hedging transactions, regardless of whether participant P wins the championship, the company’s promotional economic benefits, implicit benefits and total revenue are the same. The company neither suffers extreme losses due to contestant P winning the championship, nor obtains large excess returns due to P losing the championship, consequently avoiding to some extent the risks of uncertainty and extreme loss. In order to present the total income of the enterprise whether P wins the championship or not is uniformly recorded as TR, and the formula (1) is substituted into the TR expression,

$$TR=-\alpha bp_{0}^{2}r^{2}+[(bs-{\alpha Q}_{0})p_{0}+d]r$$ (7)

When companies plan promotional activities, they generally expect maximum social visibility and brand value through promotional activities to obtain the greatest implicit benefits, but they also minimize the direct economic losses caused by promotional activities and minimize promotional costs. Accounting for this, companies should plan promotional activities based on two promotional goals: 1) a promotional plan based on the promotional cost expenditure-implicit income balance goal, selecting an appropriate refund rate r, so that the implicit benefits (from increasing social awareness and brand value) offset or even exceed the direct economic losses of the promotion; 2) a promotional plan based on maximizing the total revenue target and an appropriate refund rate r to maximize the company’s total revenue from promotional activities (including explicit economic benefits and implicit benefits).

#### Option hedging promotion scheme based on promotion cost expenditure-implicit income balance target (decision objective 1)

From *TR*≥0, we can get $0\leq r\leq \frac{(bs-{\alpha Q}_{0})p_{0}+d}{\alpha bp_{0}^{2}}$. In the practice of corporate promotional activities, the refund rate is generally between 0 and 1. Furthermore, in order to attract more consumers to participate in promotional activities to obtain the largest possible social visibility, companies often choose the possible highest refund rate.

From $\frac{(bs-{\alpha Q}_{0})p_{0}+d}{\alpha bp_{0}^{2}}\geq 1$, we can get $bp_{0}s+d\geq \alpha bp_{0}^{2}+{\alpha p_{0}Q}_{0}$. That is, when $bp_{0}s+d\geq \alpha bp_{0}^{2}+{\alpha p_{0}Q}_{0}$, the promotion refund rate r = 1.

From $\frac{(bs-{\alpha Q}_{0})p_{0}+d}{\alpha bp_{0}^{2}}\leq 0$, we can get $bp_{0}s+d\leq {\alpha p_{0}Q}_{0}$. That is, when $bp_{0}s+d\leq {\alpha p_{0}Q}_{0}$, the promotion refund rate r = 0.

From $0<\frac{(bs-{\alpha Q}_{0})p_{0}+d}{\alpha bp_{0}^{2}}<1$, we can get ${\alpha p_{0}Q}_{0}<bp_{0}s+d<\alpha bp_{0}^{2}+{\alpha p_{0}Q}_{0}$. That is, when ${\alpha p_{0}Q}_{0}<bp_{0}s+d<\alpha bp_{0}^{2}+{\alpha p_{0}Q}_{0}$, the promotion refund rate is $r=\frac{(bs-{\alpha Q}_{0})p_{0}+d}{\alpha bp_{0}^{2}}$, which is between 0 and 1.

#### Option hedging promotion scheme based on the goal of maximizing total income (decision objective 2)

From $\frac{\partial TR}{\partial r}=0$, we can get $r=\frac{(bs-{\alpha Q}_{0})p_{0}+d}{2\alpha bp_{0}^{2}}$.

Using the same method as above, when $bp_{0}s+d\geq 2\alpha bp_{0}^{2}+{\alpha p_{0}Q}_{0}$, the promotion refund rate is r = 1; when $bp_{0}s+d\leq {\alpha p_{0}Q}_{0}$, the promotion refund rate is r = 0; when ${\alpha p_{0}Q}_{0}<bp_{0}s+d<2\alpha bp_{0}^{2}+{\alpha p_{0}Q}_{0}$, the promotion refund rate is $r=\frac{(bs-{\alpha Q}_{0})p_{0}+d}{2\alpha bp_{0}^{2}}$, which is between 0 and 1.

**Fig 1:**
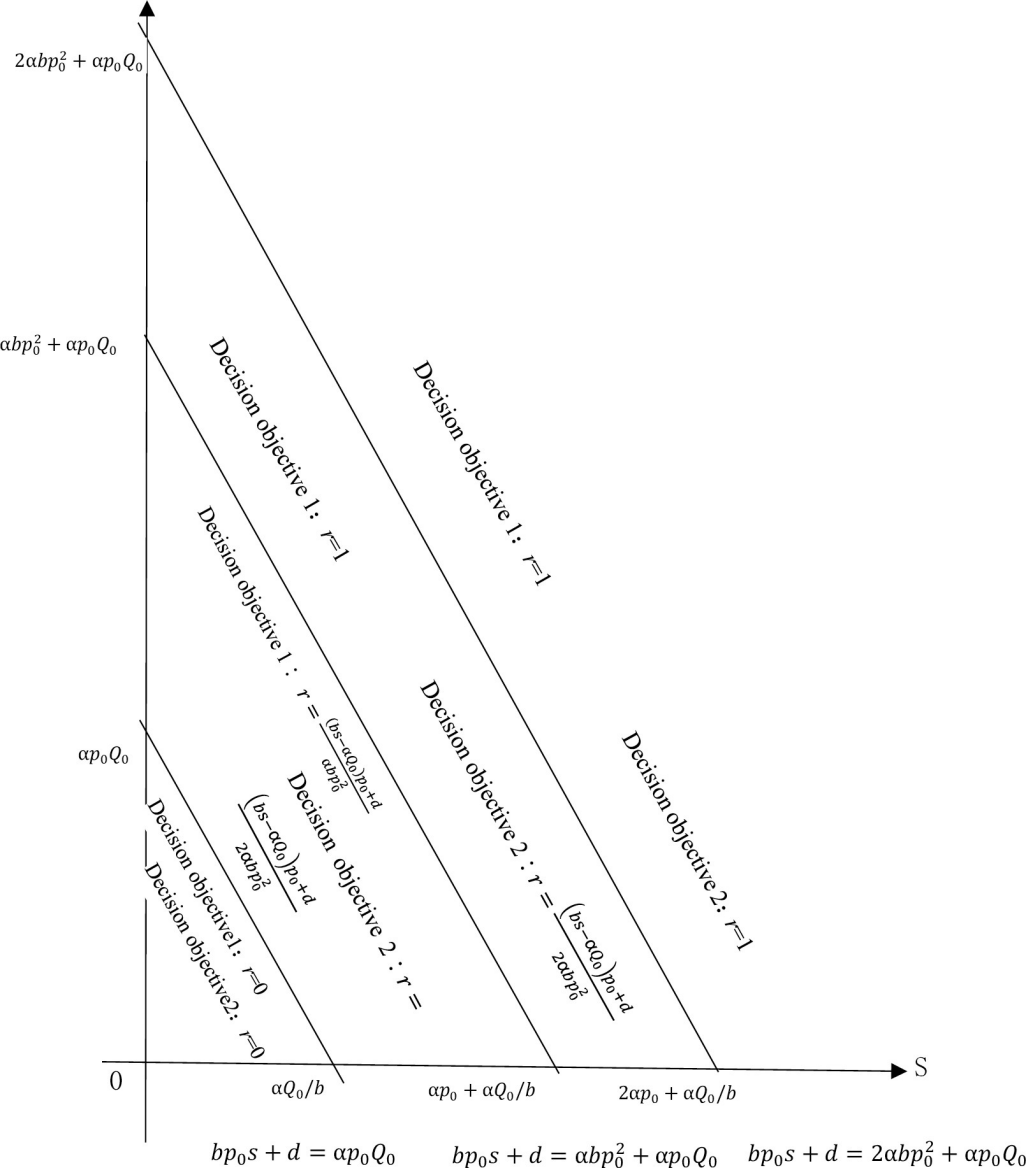
Schematic diagram of refund rate decision of the enterprise promotion scheme under two different decision goals.

Based on the above analysis, (Fig 1) is a schematic diagram of the refund rate decision of the enterprise promotion scheme under the two different decision goals.

From this, the following conclusions can be drawn:

When a company uses a large-scale sports event to carry out promotion activities and conducts option hedging transactions in the sports betting market, the gambling company sets the winning odds A0, and odds reduction factor α, as well as the sales volume Q_0_ without any promotion activities of designed products. Moreover, under the condition of participant P winning the championship with the promotion refund rate of 100%, the refund rate r depends on the return on sales and the maximum promotional implicit income (or the maximum promotional expense the company is willing to pay) d, the decision of the refund rate is demonstrated as follows:

1. When the company’s return on sales s and the maximum implicit income d from promotional activities satisfies $bp_{0}s+d\leq {\alpha p_{0}Q}_{0}$, the promotional plan based on the balance objective of promotion cost expenditure and implicit income, as well as the refund rate of maximizing total revenue is 0 (r = 0); the company chooses no promotion activities.
2. When ${\alpha p_{0}Q}_{0}<bp_{0}s+d<\alpha bp_{0}^{2}+{\alpha p_{0}Q}_{0}$, the promotion plan based on the promotion cost expenditure-implicit income balance goal and maximizing the total promotion revenue, the refund rates are $r=\frac{(bs-{\alpha Q}_{0})p_{0}+d}{\alpha bp_{0}^{2}}$ and $r=\frac{(bs-{\alpha Q}_{0})p_{0}+d}{2\alpha bp_{0}^{2}}$, which are between 0 and 1; the company chooses different partial refund promotions.
3. When $\alpha bp_{0}^{2}+{\alpha p_{0}Q}_{0}<bp_{0}s+d<2\alpha bp_{0}^{2}+{\alpha p_{0}Q}_{0}$, the refund rate of the option hedging promotion plan based on the promotion cost expenditure-implicit benefit balance objective is r = 1, and the company chooses to fully refund, the option hedging promotion plan based on the goal of maximizing the total revenue with a refund rate $r=\frac{(bs-{\alpha Q}_{0})p_{0}+d}{2\alpha bp_{0}^{2}}$; the company chooses a partial refund promotion plan.
4. When $bp_{0}s+d\geq 2\alpha bp_{0}^{2}+{\alpha p_{0}Q}_{0}$, the refund rates of the promotion plan based on the promotion cost expenditure-implicit income balance goal and the target of maximizing total revenue are 1 (r = 1); the company chooses a full refund promotional plan.

### Analysis of the effect of corporate promotion risk management and control based on option hedging transactions

#### Uncertainty risk management and control: Return-variance analysis

Eqs (3) and (4) indicate that the expected total return and variance of the promotional activities without option hedging transactions are, respectively:

$$E({TR}_{0})=(Q-Q_{0})s+dr-Qr∙P_{0};Var({TR}_{0})=Q^{2}r^{2}p_{0}(1-p_{0})$$

In the case of using option hedging transactions, the expected total return and variance of the enterprise are, respectively:

$$E(TR)=(Q-Q_{0})s+dr-Qr∙\alpha p_{0};\;Var(TR)=0$$

Obviously, Var $\left({TR}_{0})>Var(TR);\;E({TR}_{0})-E(TR)=Qrp_{0}(\alpha -1\right)$. Since the odds reduction factor α is greater than 1, so E(*TR*_0_)>E(*TR*). This is consistent with the principle of risk-return equivalence in modern financial market theory. When companies carry out promotional activities, it is possible to reduce risk by giving up part of the expected total revenue, thereby maximizing utility. Assuming that the enterprise is risk averse and its risk aversion coefficient is ρ, the utility of the enterprise without and with option hedging transactions are, respectively:

$$U({TR}_{0})=E({TR}_{0})-\frac{1}{2}\rho Var({TR}_{0})=(Q-Q_{0})s+dr-Qr∙p_{0}-\frac{1}{2}\rho Q^{2}r^{2}P_{0}(1-p_{0})$$

$$U(TR)=E(TR)-\frac{1}{2}\rho Var(TR)=(Q-Q_{0})s+dr-Qr∙\alpha p_{0}$$

$$U(TR)-U({TR}_{0})=Qrp_{0}(1-\alpha )+\frac{1}{2}\rho Q^{2}r^{2}p_{0}(1-p_{0})$$

From U(TR)−U(*TR*_0_)≥0, we can get $\rho \geq \frac{2(\alpha -1)}{Qr(1-p_{0})}$. That is, when $\rho \geq \frac{2(\alpha -1)}{Qr(1-p_{0})}$, U(TR)≥U(*TR*_0_). To sum up, expected benefits and risks when using option transactions for hedging are smaller than when not using them. Under the condition that the enterprise’s risk aversion is higher than a certain threshold, an option hedging transaction can not only reduce the risk to zero, but also obtain a level of utility not lower than that without option hedging. This shows that when companies use large-scale sports events to carry out promotional activities, option hedging transactions can effectively manage uncertain risks.

#### Analysis of extreme losses risk management and control

When companies use large-scale sports events to carry out promotional activities, they focus not only on the expected benefits and comprehensive utility, as well as the uncertain risks, but also on the risk of extreme losses when adverse events occur.

Comparing the promotion plan with and without the hedging transaction option, it can be seen that the promotion plan without the hedging transaction option may incur extreme losses of *Qr*−(*Q*−*Q*_0_)*s*−*dr*, when contestant P wins. However in the promotion scheme with the hedging transaction option, this loss reduces to $\frac{Qr}{A_{0}}-(Q-Q_{0})s-dr$, and the reduction range is $(1-\frac{1}{A_{0}})Qr$. Based on the above assumptions, $\left(1-\frac{1}{A_{0}}\right)Qr>0$. This shows that the hedging transaction option reduces extreme losses for a company when contestant P wins the championship, and thus effectively manages the extreme loss risk in the promotion of large-scale sports events.

## A case analysis based on the promotion of Vatti shares’ 2018 World Cup "If France Wins, Get a Full Refund"

Vatti was established in 2001 with a registered capital of 872.643124 million yuan. Its predecessor was Zhongshan Huadi Gas Appliance Co., Ltd., established in 1992, and Vatti’s shares were listed on the Shenzhen Stock Exchange in September 2004. In over 10 years since its public listing, Vatti has achieved a series of impressive results. According to the company’s official website, Vatti gas cookers ranked first in the sales of similar products in the national market for 14 consecutive years from 1996 to 2009. Vatti was awarded the title of “Group Member Unit of China Association for Quality Inspection” in 2010, one of the “Top Ten Excellent Independent Brands of China Household Electrical Appliances Industry Trusted by Global Consumers” in 2011, and awards for contract-honoring and credit-respecting enterprise in Guangdong province for 19 consecutive years up to 2014. Vatti was awarded the core technology and innovation award in 2015, and the “International Industry Impact Brand Award” from the United Nations in May 2016. In March 2017, the magic dish stove product won the German IF Award, the German Red Dot Award and the Bronze Award of American IDEA Design. In December 2017, it was listed on the Top 500 Chinese Brands Value. At the same time, Vatti has made outstanding achievements in technological innovation, launching its first robot V in the field of kitchen and electrical appliances in August 2016, and successfully entering into the field of artificial intelligence. Up to May 10, 2018, Vatti had authorized 838 effective patents including Vatti tobacco machine voice control, flying saucer type turning furnace head, water heater temperature control cabin, steaming water washing, stove gathering energy combustion amongst its core innovations.

### The promotion process of Vatti in the 2018 World Cup “If France Wins, Get a Full Refund”

The 2018 Russia Football World Cup was held from June 14th (t1) to July 16th (T), 2018. Vatti Co., Ltd. (referred to as Vatti shares, stock code 002035), the official contract partner and sponsor of the French National Football Team planned the promotion activity of "If France Wins, Get a Full Refund".

On May 30, 2018, Vatti shares issued an announcement stating that if the French team won the 2018 World Cup in Russia, consumers who bought the “champion package” from 0:00 (t0) on June 1, 2018 to 22:00 on June 30, 2018 would get a full refund. The French team defeated the Argentine team 4:3 and entered the quarter finals. Vatti shares announced the "If France Wins, Get a Full Refund" event would be extended for three days to 22:00 on July 3 (t2).

On July 4, 2018, Vatti preliminary statistics stated that the offline channel sales of the designated products of the “champion package” from 0:00 on June 1, 2018 (t0) to 22:00 on July 3 (t2) was approximately 50 million yuan, online channel sales were about 29 million yuan, and total online and offline sales were about 79 million yuan. On July 16, 2018, following the French win of the championship, Vatti issued a statement regarding the launch of the refund process for the "If France Wins, Get a Full Refund "event.

### Analysis of the promotional effect of Vatti shares’ 2018 World Cup “If France Wins, Get a Full Refund"

Following the French win of the championship, Vatti shares became famous for betting on the French team in the championship. Its "If France Wins, Get a Full Refund " campaign aroused widespread public attention and response. It improved the brand of Vatti Corporation, and significantly increased Vatti’s online and offline sales. On the surface it achieved a positive advertising effect. According to Vatti Shares, during the "If France Wins, Get a Full Refund" event, total offline sales were above 700 million yuan, a year-on-year increase about 20%. The offline sales of the "champion package" product (50 million yuan) accounted for about 7% of the company’s offline channel sales, and online channel sales were more than 300 million yuan, an increase of about 30% year-on-year. The online sales of "If France Wins, Get a Full Refund" accounted for 9.67% of the company’s online channel sales.

If Vatti shares had been able to fulfill its promise and unconditionally return the sales of 79 million yuan of the "champion package" products to consumers, it would have had a very positive effect on Vatti shares’ future market expansion, brand image establishment, and business performance improvement. However, unfortunately, during the refund process of the "If France Wins, Get a Full Refund" promotion, Vatti passed on the offline sales refund obligation to regional distributors and set various restrictions and conditions on refunds to consumers who had bought the champion package online and offline. This resulted in criticism of Vatti from both consumers and the public, negating the previously formed advertising effect and damaging the company’s image.

Why did Vatti mess up the "If France Wins, Get a Full Refund" campaign? Why did Vatti fail to use the event to establish premium image and industry status? Why self-destroy its image and public goodwill after the event? The following three reasons apply: Firstly, the uncertainty and extreme loss risks generated by the "If France Wins, Get a Full Refund" campaign had not been accurately assessed; secondly, the adverse situation had not been worked out in response to an extreme loss risk strategy and program; thirdly, there was no effective management and control of risk management tools in the financial market, especially in the financial derivatives market. The serious flaws in the design of Vatti’s campaign for the "If France Wins, Get a Full Refund" was instrumental in its failure to achieve the expected promotional effect. The risk management tools of the financial derivatives market could provide methods and approaches for Vatti’s control of the uncertainty and extreme loss risks in promotional activities.

### Improvement of the promotional plan of Vatti shares’ 2018 World Cup "If France Wins, Get a Full Refund" based on option hedging theory

This section intends to use the corporate promotion risk management model based on the option hedging theory constructed in the second section to improve the promotion plan of Vatti shares’ 2018 World Cup "If France Wins, Get a Full Refund" campaign.

Firstly, determination of the probability p_0_ of the French team winning the 2018 World Cup in Russia and the odds reduction factor α.

The bookmaker’s World Cup winning odds for the French team on June 1, 2018 was 7.5 to 1, with the odds reduction factor set to 1.2. The parameters in the second quarter model are A_0_ = 7.5, α = 1.2, p_0_ = 1/9.

Secondly, the normal sales on the designated products of champion package without promotional activity is Q0, the maximum promotional amount is b when the French team wins the championship (p0 = 1) and Vatti fully refunds (r = 1). This determines the functional relationship between the total promotion amount of the designated product of the "champion package" during the promotional period and the refund rate r as well as the French winning probability p0.

As Vatti shares did not announce the year-on-year increment in sales of its "champion package" products during the promotion period, this paper uses only the total sales growth range for calculation, due to data availability. According to the announcement of Vatti Co., Ltd, during the "If France Wins, Get a Full Refund" event, the total offline channel sales of Vatti were above 700 million yuan with a year-on-year increase of about 20%, and online channel sales were about 300 million yuan with an increase of about 30% year-on-year. The sales growth range of its "champion package" should be greater than that of the non-"champion package" designated products. Assuming that sales of the offline and online channels of Vatti’s "champion package" designated products have increased about 40% and 50% year-on-year, the offline and online channel sales of the designated product of the "champion package" in 2017 were 35 million yuan and 19 million yuan, respectively. From this, Q0 = 5400 can be set. Since the total sales (Q = 7900) of the designated products of the "champion package" during the promotional period comes from the condition of a full refund (refund rate r = 1) according to formula (1), b = 22500, the functional relationship between the total promotion amount of the "champion package" product during the promotion period and the refund rate r as well as the French win probability p0 can be expressed as

$$Q=5400+22500rp_{0}$$ (8)

**Fig 2:**
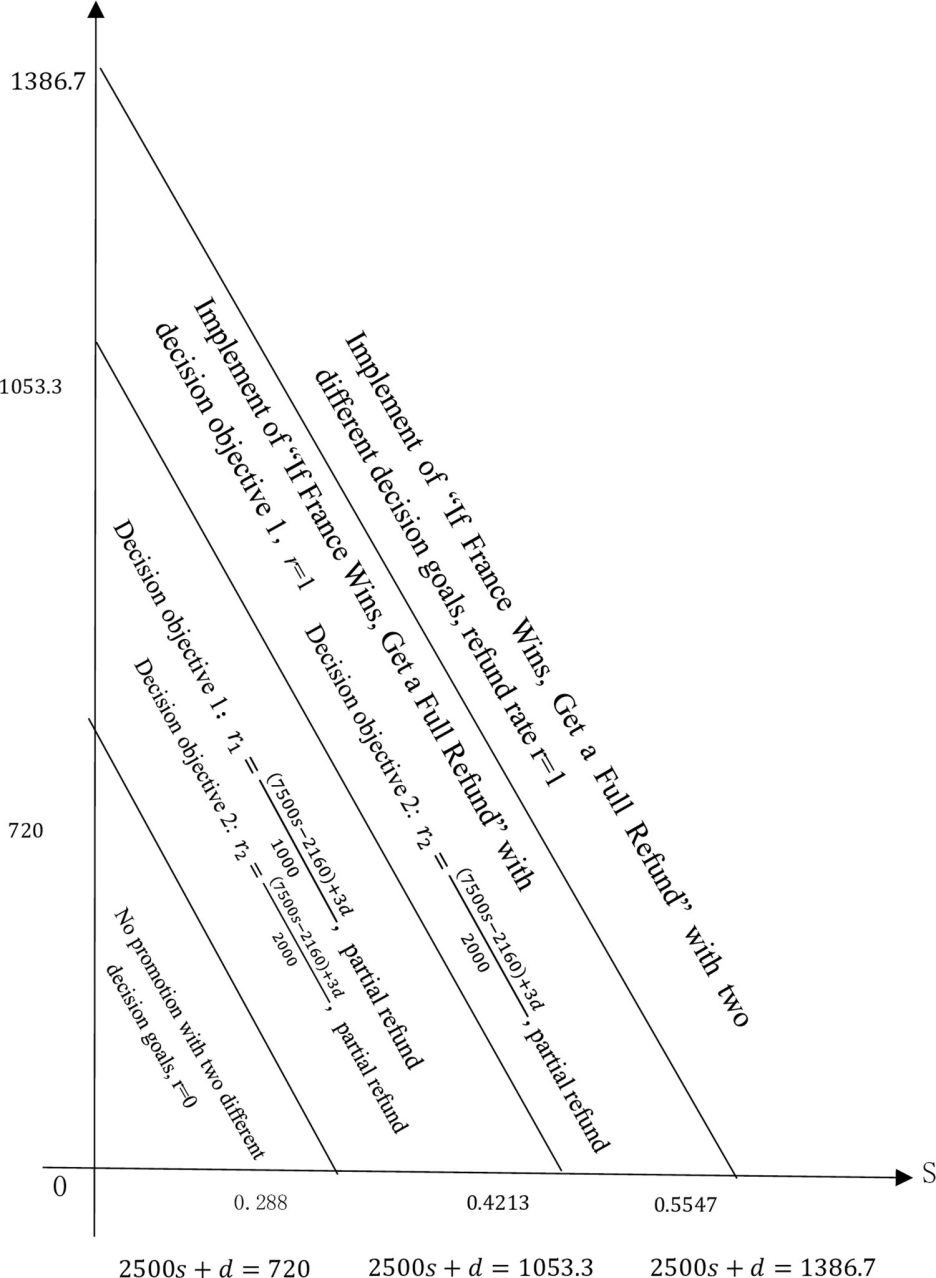
Schematic diagram of the promotion plan of Vatti shares in the 2018 World Cup under two different decision goals.

Thirdly, determination of the promotion plan of Vatti shares based on option hedging transactions. Based on the parameters A0, α, p0, Q0, and b determined in the above steps and the theoretical model in Section 2, Vatti shares’ "champion package" promotion plan is shown in (Fig 2).

According to Vatti’s annual report from 2010 to 2017, it can be estimated that the average return on sales s is 7.5%. In the absence of major changes at the technical level and in the operating environment, the company’s return on sales remains relatively stable. Therefore, the promotional plan for the 2018 World Cup should follow the maximum promotional fee, which is willingness to pay or obtain the maximum implicit benefits (social visibility or brand value) change to change. When d ≤ 532.5, the maximum promotional fee that the company is willing to pay is less than 5.325 million yuan, and the refund rate of the promotion plan based on the promotion cost expenditure-implicit benefit balance goal and the goal of maximizing total revenue are both 0, Vatti shares does not carry out promotional activities. When d ≥ 1199.2, the maximum promotion fee that the company is willing to pay is more than 11.992 million yuan, and the refund rate for the promotion plan based on the promotion cost expenditure-implicit income balance goal and maximizing total revenue is 1, Vatti shares should fully refund. When 532.5 < d < 865.8, the maximum promotional expenses that the company is willing to pay are between 5.325 million yuan and 8.658 million yuan, based on the promotion cost expenditure-implicit income balance goal and maximizing total revenue, and the refund rates are $r=\frac{3d-1597.5}{1000}$ and $r=\frac{3d-1597.5}{2000}$, Vatti shares uses a partial refund promotion plan with different refund rates. When 865.5 < d < 1199.2, the maximum promotional expense that the company is willing to pay is between 8.658 million yuan and 11.992 million yuan based on the promotion cost expenditure-implicit income balance target, the selected refund rate r = 1, Vatti shares adopts the full refund promotion plan. The refund rate of the promotion plan based on maximizing total revenue is $r=\frac{3d-1597.5}{2000}$ and Vatti shares uses the partial refund promotion plan. Vatti shares only needs to make appropriate promotion decisions and promotion plans based on the maximum promotional expenses it is willing to pay or the maximum implicit benefits of social visibility or brand value.

### Analysis of the risk control effect of the Vatti Shares’ promotion plan based on option hedging transactions

#### Promotional risks and extreme losses of Vatti shares’ current promotion plan

In June 2018, Vatti shares launched its "If France Wins, Get a Full Refund" promotion, setting the refund rate r = 1, but there was no option hedging transaction in the gaming market. The total additional revenue from promotions under the conditions of the French team either losing and winning the championship are respectively

$${TRN}_{0}=187.5+d;\;{TRY}_{0}=-7712.5+d$$

The expected return, variance and utility levels are:

$$E({TR}_{0})=-6212.5/9+d;\;Var({TR}_{0})=6163950.6;\;U({TR}_{0})=-6212.5/9-3081975.3\rho +d$$

From the above analysis, it can be seen that Vatti shares would get an additional promotion income of 187.5 + d (including an economic income of 1.875 million yuan) if the French team lost the championship, while it would suffer -7712.5 + d additional promotion loss (77.125 million of economic losses among them) if the French team won the championship. If the annual budget promotion expenses of Vatti shares or the maximum implicit income (public awareness or brand value) is less than 77.125 million yuan, then the promotion expenses of Vatti shares will exceed the budget or the implicit income will struggle to offset the direct economic loss. Consequently, Vatti transferred the offline sales refund obligation to regional distributors and set various restrictions and conditions on the online and offline "champion package" consumer refunds, thereby swindling consumers and damaging their reputation.

#### Promotional risks and extreme losses in Vatti’s promotion plan based on option hedging theory

By using option hedging transactions, the expected total return, variance, and utility level of Vatti shares are respectively:

$$E(TR)=-532.5r-\;\frac{1000}{3}r^{2}+dr;\;Var(TR)=0;\;U(TR)=-532.5r-\frac{1000}{3}r^{2}+dr$$

It is clear that when using option hedging transactions, Vatti shares can not only reduce the promotion risk to zero, but also avoid extreme loss risks.

#### Risk management and control effect of Vatti’s promotion plan for the 2018 Football World Cup based on option hedging transactions

Table 1 demonstrates the risk management and control effect of Vatti’s promotion plan for the 2018 Football World Cup based on option hedging transactions. It shows that under the condition of a certain return on sales of Vatti shares (7.5%), when the maximum implicit income (the largest annual promotion expense that the company is willing to pay) is relatively small (d ≤ 532.5), the current promotion plan produces negative expected total revenue and total utility, with great uncertainty and extreme loss risks. The option hedging promotion plan, based on a promotional cost expenditure-implicit revenue balance goal and maximizing the total revenue target, has effectively avoided these potential losses and risks by not carrying out promotional activities.

**Table 1:** Risk management and control effect of Vatti shares promotion plan based on option hedging transaction.

Maximum implicit revenue (the maximum promotional fee the company is willing to pay) d	Promotional plan		Refund rate r	France team wins the champion or not	Total revenue	Extreme losses	Expected income	Variance	Utility	Comparative results
d ≤ 532.5	Plan 1		Promotion with full refund (r = 1)	France team loses championship	(187.5, 720)	(7180, 7712.5)	(-690.3, -157.8)	6163950.6	(-690.3–3081975.3ρ, -157.8–3081975.3ρ)	The option hedging promotion scheme based on promotional cost expenditure-implicit income balance objective and maximizing total income objective is better.
				France team wins championship	(-7712.5, -7180)					
	Plan 2	Plan 21	No promotion (r = 0)	France team loses championship	0	No losses	0	0	0	
				France team wins championship	0					
		Plan 22	No promotion (r = 0)	France team loses championship	0	No losses	0	0	0	
				France team wins championship	0					
532.5 < d < 865.8	Plan 1		Full refund (r = 1)	France team loses championship	(720, 1053.3)	(6846.7, 7180)	(-157.8,175.6)	6163950.6	(-3081975.3(ρ+0.000051), -3081975.3(ρ-0.000057))	The option hedging promotion plan based on maximizing total revenue is the best, and the option hedging promotion plan based on the promotional cost expenditure-implicit income balance goal is second best; both are better than the current promotion plan.
				France team wins championship	(-7180, -6846.7)					
	Plan 2	Plan 21	$r=\frac{3d-1597.5}{1000}$ Promotion with partial refund (0 ≤ r ≤ 1)	France team loses championship	0	No losses	0	0	0	
				France team wins championship	0					
		Plan 22	$r=\frac{3d-1597.5}{2000}$ Partial refund (0 ≤ r ≤ 0.5)	France team loses championship	(0, 83.4)	No losses	(0, 83.4)	0	(0, 83.4)	
				France team wins championship	(0, 83.4)					
865.8 < d < 1199.2	Plan 1		Promotion with full refund (r = 1)	France team loses championship	(1053.3, 1386.7)	(6513.3, 6846.7)	(175.6, 508.9)	6163950.6	(-3081975.3(ρ-0.000057), -3081975.3(ρ-0.000165))	The option hedging promotion plan based on maximizing total revenue is the best, and the option hedging promotion plan based on the promotional cost expenditure-implicit income balance goal is second best; both are better than the current promotion plan
				France team wins championship	(-6846.7, -6513.3)					
	Plan 2	Plan 21	Promotion with full refund (r = 1)	France team loses championship	(0, 333.4)	No losses	(0, 333.4)	0	(0, 333.4)	
				France team wins championship	(0, 333.4)					
		Plan 22	$r=\frac{3d-1597.5}{2000}$ Promotion with partial refund (0.5 ≤ r ≤ 1)	France team loses championship	(83.4, 333.4)	No losses	(83.4, 333.4)	0	(83.4, 333.4)	
				France team wins championship	(83.4, 333.4)					
d ≥ 1199.2	Plan 1		Promotion with full refund (r = 1)	France team loses championship	1386.7 + (d-1199.2)	6513.3-(d-1199.2)	508.9+(d-1199.2)	6163950.6	-3081975.3(ρ-0.000165) +(d-1199.2)	The option hedging promotion scheme based on promotional cost expenditure-implicit income balance objective and maximizing the total income objective is better.
				France team wins championship	-6513.3 + (d-1199.2)					
	Plan 2	Plan 21	Promotion with full refund (r = 1)	France team loses championship	333.4 + (d-1199.2)	No losses	333.4+(d-1199.2)	0	333.4+(d-1199.2)	
				France team wins championship	333.4 + (d-1199.2)					
		Plan 22	Promotion with full refund (r = 1)	France team loses championship	333.4 + (d-1199.2)	No losses	333.4+(d-1199.2)	0	333.4+(d-1199.2)	
				France team wins championship	333.4 + (d-1199.2)					

Notes: Plan 1: Current promotion plan with full refund and no option hedging. Plan 2: promotion plan based on option hedging. Plan 21: the option hedging promotion plan based on promotion cost expenditure-implicit income balance objective. Plan 22: the option hedging promotion plan based on maximizing total income objective.

When the maximum implicit income d is between 532.5 and 865.8, the current promotion plan still causes Vatti shares to suffer great uncertainty and extreme loss risk. Although when the maximum implicit income increases from 532.5 to 865.8, the expected return turns from negative to positive, as long as Vatti shares closes its risk aversion (ρ > 0.000057), the utility is always negative. Based on a promotional cost expenditure-implicit revenue balance goal and maximizing the total revenue target, the risk aversion of Vatti shares avoids the uncertainty risk and extreme losses by setting a reasonable refund rate (partial refund) and conducting option hedging transactions, achieving relatively higher utility. Moreover, the option hedging promotion plan based on the goal of maximizing the total income shows better promotional effects.

When the maximum implicit income d is between 865.8 and 1199.2, although the current promotion plan enables Vatti shares to obtain positive expected returns, it also suffers great uncertainty risk and extreme losses. Furthermore, if Vatti shares closes its risk aversion (ρ > 0.000165), its utility is always negative. Vatti does not undertake uncertainty risks and extreme losses based on a promotional cost expenditure-implicit revenue balance goal and maximizing the total revenue target, it gives full or partial refunds as well as obtaining higher expected returns and utility by option hedging transactions. Moreover, the option hedging promotion scheme based on the goal of maximizing total revenue also shows a better promotional effect.

When the maximum implicit income d is relatively large (d ≥ 1199.2), both the current promotion plan and the option hedging promotion plan should adopt the full refund method of "If France Wins, Get a Full Refund". In comparison with the promotion plan based on option hedging transactions, Vatti undertakes a greater uncertainty risk and extreme losses. Although the current promotional plan should obtain a higher expected return than that based on option hedging transactions, as long as Vatti shares closes its risk aversion, the level of utility should be lower than the promotion plan based on option hedging transactions. Moreover, the option hedging promotion program shows the same promotional effect based on the promotional cost expenditure-implicit income balance objective and the goal of maximizing the total return.

From the above analysis, the option hedging promotion plan based on the goal of maximizing total revenue shows the best promotional effect, and the option hedging promotion plan based on the promotional cost expenditure-implicit income balance goal is the second best; both are better than the current promotion plan.

According to Vatti shares, the company’s promotional fee was 455 million yuan in 2017. During the "If France Wins, Get a Full Refund" campaign, the offline sales (50 million yuan) of Vatti’s “champion package” accounted for approximately 7% of the company’s offline channel sales (around 700 million yuan). Online sales (29 million yuan) accounted for approximately 9.67% of the company’s online sales (about 300 million yuan). The proportion of the "champion package" of the company’s total sales without promotion activities should be much lower than that with promotional activities. It is assumed that the sales of "champion package" products without promotional activities accounted for 4% of the company’s total sales, and the promotional cost of the "champion package" product that Vatti was willing to undertake was 455 million yuan * 4% = 18.2 million yuan, that is, d = 1820 > 1199.2. Under the condition of option hedging transactions, Vatti shares should launch the "If France Wins, Get a Full Refund" promotion plan. Based on this, under the condition of the maximum implicit income of 18.2 million yuan, the risk control effect of Vatti’s option hedging promotion plan relative to the current promotion plan (as shown in Table 2) can be calculated. It can be seen from Table 2 that if the implicit income or the maximum promotional expenses the company is willing to pay is d = 1820, then the option hedging promotion plan based on the promotional cost expenditure-implicit income balance objective, as well as maximizing the total income as the target, show the same promotional effect. Both of these are better than the current promotion plan. Therefore, it would have been reasonable to use a full refund plan for the promotion of Vatti shares in the 2018 World Cup "If France Wins, Get a Full Refund". The main problem was that the derivatives financial instruments provided by the sports betting market were not used in promotional activities for option hedging transactions to control promotional risk. Regardless of whether Vatti had adopted an option hedging promotion plan based on the promotional cost expenditure-implicit income balance goal, or the target of maximizing total revenue, its 2018 World Cup "If France Wins, Get a Full Refund" promotion should have completely avoided the risk of uncertainty and extreme losses, gaining positive returns as well as utility. It should not have caused Vatti to pass the obligation of offline sales refunds to regional distributors and to set various restrictions and conditions for refunding the "champion package" online and offline after the French team won the championship. This would have come with an expected positive promotional effect.

**Table 2:** The risk management and control effect of the Vatti shares promotion plan based on option hedging transactions under the condition of maximum implicit income, *d* = 1820.

Promotion plan		Refund rate *r*	France team wins the champion or not	Total revenue	Extreme losses	Expected income	Variance	Utility	Comparative results
Current promotion plan		Full refund (r = 1)	France team loses championship	2007.5	5892.5	1129.7	6163950.6	-3081975.3 (ρ - 0.000057) + 954.2	The option hedging promotion plan based on maximizing total revenue is the best, and the option hedging promotion plan based on the promotional cost expenditure-implicit income balance goal is the second best; both are better than the current promotion plan.
			France team wins championship	-5892.5					
Promotion plan based on the option hedging theory	the option hedging promotion plan based on promotional cost expenditure-implicit income balance objective	Full refund (r = 1)	France team loses championship	954.2	With gains, without losses	954.2	0	954.2	
			France team wins championship	954.2					
	the option hedging promotion plan based on maximizing the total income objective	Full refund (r = 1)	France team loses championship	954.2	With gains, without losses	954.2	0	954.2	
			France team wins championship	954.2					

## Conclusions

The use of large-scale sports events to carry out promotional activities has become an important way for companies to enhance social visibility and brand value; however, most companies do not use derivative financial instruments provided by the sports betting market to effectively control promotion risks in promotional activities. As a result, companies can face risks of uncertainty and extreme losses beyond their ability to bear. This not only fails to achieve the expected promotional effect, but may also harm the corporate image and brand value. The process and results of the promotional activities of Vatti shares in the 2018 Football World Cup "If France Wins, Get a Full Refund" promotion, highlight the importance of corporate promotion risk management and control, and provides a classic case and lessons for companies using large-scale sports events to carry out promotional activities.

On the basis of an in-depth investigation of the practice of companies using large-scale sports events to carry out promotional activities, and of the derivative financial instruments provided by the sports gambling market, this paper constructs a risk control model for companies using large-scale sports events for promotional activities, based on option hedging theory. The paper proposes an option hedging promotion plan based on the cost expenditure-implicit income balance objective and maximizing total income. The research results show that when the winning odds offered by the bookmaker for the participants, the odds reduction factor, and the normal sales of the company’s designated products without promotional activities are fixed, as well as the company’s P100% of the participants and the refund rate 100% also being fixed; then the refund rate of corporate promotional activities depends on the company’s return on sales and the maximum promotional implicit revenue (or the maximum promotional fee willing to pay). Specifically, when the company’s return on sales and the maximum implicit revenue generated by promotional activities are relatively small (the combination is less than a certain threshold), the company should not choose to carry out promotional activities. When the company’s return on sales and the maximum implicit revenue generated by promotional activities are neither too high nor too low (the combination is between two certain thresholds), the company should choose a partial refund promotion plan. When the company’s return on sales and the maximum implicit revenue generated by promotional activities are high (the combination is greater than a certain threshold), the company should choose a promotion plan with a full refund. When companies use large-scale sports events for promotional activities, they should carry out option hedging transactions to effectively manage uncertain risks and extreme loss potential.

From the analysis of the process and results of the activities of Vatti shares’ 2018 Football World Cup "If France Wins, Get a Full Refund" promotion, this article uses the constructed promotion risk management model based on the option hedging theory to control Vatti shares’ 2018 World Cup promotional campaign. A case analysis and program improvement were carried out for the promotion campaign of "If France Wins, Get a Full Refund". The case study results show that it would have been reasonable to use a full refund plan for the Vatti shares’ promotion, through the option hedging plan and the promotional cost expenditure-implicit income balance objective, and maximizing the total income target. This should have enabled Vatti to obtain positive returns and utility while avoiding the risks of uncertainty and extreme losses. Both practices show the same positive promotional effect, superior to the actual promotion program (without option hedging transactions).

The research paper applies risk management tools of the financial derivatives market to the practice of corporate promotion risk management and control, and provides effective solutions and practical cases of derivative financial instruments for companies to effectively control uncertainty and extreme loss risks in promotions involving large-scale sports events. It opens a new research field of derivative financial instruments and corporate promotion risk management.

The research work in this article is only a phasic research result of the author’s research on “derivative financial instruments and corporate promotion risk management” and there are many related issues that need to be studied further. For example, in consideration of the sales of promotional products affected by the external macro environment, further research issues include: the model and strategy of using derivative financial instruments to control corporate promotion risk; selecting the model and strategy of the optimal time point for using derivative financial instruments to hedge transactions during the promotion of large-scale sports events; and using the model and strategy of corporate promotion risk management and control, based on a combination of multiple derivative financial instruments and hedging transactions in the promotion of large-scale sports events. The author intends to focus on these issues in subsequent research.

The research paper is only a phasic research result on derivative financial instruments and corporate promotion risk management but there are many related issues that need to be studied further. From the external aspect, the module in the paper can not be used when the macro environment changes, we have to consider about other factors to affect the strategy. From the inside, the enterprise could utilize the portfolio hedging transactions so that the module will be changed. Moreover, the author intends to expand the sample not only the special case, but also other enterprises and improve the practical significance. These issues will be focused on subsequent research.
